# When AI takes the wheel: AI-defined vehicles principles and pitfalls

**DOI:** 10.3389/frobt.2026.1770121

**Published:** 2026-03-02

**Authors:** Marco De Vincenzi, Chiara Bodei, Ilaria Matteucci

**Affiliations:** 1 Institute of Informatics and Telematics - CNR, Pisa, Italy; 2 Department of Computer Science, University of Pisa, Pisa, Italy

**Keywords:** artificial intelligence (AI), autonomous vehicle (AV), intelligent transportation system (ITS), safety, security, vehicle-to-everything (V2X)

## Abstract

As introduced by Asimov in “I, Robot”, intelligent machines are characterized as systems capable of performing tasks that traditionally require human intelligence, such as autonomous decision-making and driving. In this context, modern road vehicles can increasingly be understood as robotic systems endowed with progressively sophisticated functionalities, operational flexibility, and, crucially, the capacity to learn and evolve autonomously over time. Building on this perspective, AI-defined vehicles (AIDVs) are emerging in both the automotive industry and the research community as a next stage in vehicle evolution, where interaction capabilities, adaptability, sustainability, and ethical governance are embedded as core design principles rather than treated as auxiliary features. This work aims to introduce this new class of vehicles and provide an analysis of their defining principles, capabilities, and challenges. This article contributes a first conceptualization of AIDVs, outlines their defining principles, and distinguishes them from existing vehicle classes. Then, it identifies the risks introduced by adaptive AI and proposes a preliminary roadmap for their integration into Intelligent Transportation Systems (ITS).

## 1 Introduction

What if road vehicles behave like AI-driven robots? This question invites consideration of a future in which vehicle functionalities and driving behavior are no longer managed solely by software, even when augmented with AI, but instead emerge from continuous interaction with other autonomous Cyber–Physical Systems (CPSs) within a large-scale Intelligent Transportation System (ITS). In such an environment, vehicles are CPSs, and AI agents can *interact* and *autonomously make decisions* for various purposes, including driving, traffic optimization, and mobility.

Traditional software and AI differ at a fundamental level: the key distinctions lie in their operational principles, adaptability, and problem-solving capabilities. Traditional software follows explicit algorithms defined by developers. Its behavior may be deterministic or nondeterministic, e.g., due to concurrency, timing, or underspecified execution order, but it remains grounded in predefined rules and control logic, producing outcomes that are fully determined by the implemented program. AI systems, by contrast, learn directly from data, discovering patterns that guide their behavior rather than relying exclusively on handcrafted rules. Although AI is technically a form of software, it represents a fundamental shift in problem-solving: its outputs are *inherently probabilistic*, enabling adaptation to unseen situations and behaviors not explicitly programmed, while also introducing new safety challenges in CPSs due to their nondeterministic behaviors (Russell and Norvig, 2021).

**FIGURE 1:**
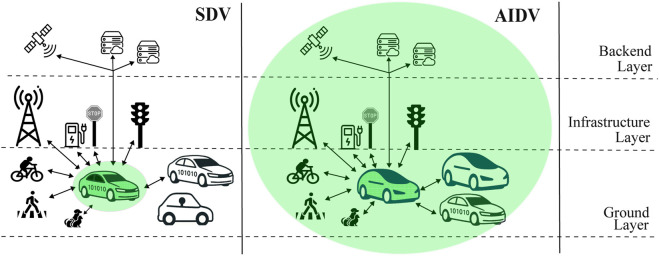
SDV and AIDV interaction *surface bubbles* across ground, infrastructure, and backend layers.

Focusing on the vehicular domain, this difference means that a vehicle is no longer a stand-alone entity interacting with its surroundings, but instead extends its operational surface to integrate with the surrounding environment, thereby defining its behavior through continuous data exchange, learning, and processing (Figure 1).

Unlike Software-defined Vehicles (SDVs), in which AI-based functions, when present, are typically confined to predefined components and operate within a fixed decision architecture, AI-defined Vehicles (AIDVs) are characterized by continuous adaptation through interaction-driven reasoning and learning, where driving and coordination policies evolve as a result of sustained interaction with other agents and infrastructure. The vehicle surface is no longer confined to the vehicle itself but extends to include interactions with other road actors, through which vehicle functionalities are enabled.

In recent years, several industry leaders have begun referring to AIDV to describe the expected evolution toward massive AI dependence in future vehicles (Che et al., 2025; Qualcomm Technologies Inc., 2026). However, these industrial references remain high-level, industry-based, and fragmented. For this reason, this work analyzes the defining principles, enabling technologies, and associated challenges of AIDVs. It consolidates and contextualizes existing industrial uses of the term, distinguishes AIDVs from conventional autonomous vehicles and SDVs, and identifies the key AI-driven capabilities that characterize this next-generation of mobility systems. Building on this foundation, the paper outlines potential risks, identifies open research questions, and discusses the requirements for the safe, trustworthy, and effective integration of AIDVs into ITSs. This work’s contributions are the following:

- Introduction of AIDV principles: The principles that distinguish AIDVs from other vehicle classes are outlined, and a reference architecture is introduced that incorporates an AI reasoning layer and digital-twin integration as core architectural invariants.
- Identification of AIDV-specific risks: Pitfalls that are specific to AIDVs are identified, e.g., emergent multi-agent behaviors, persuasive recommender effects, adversarial sensing, and overreliance on AI for security, and contrasted with known SDV risks;
- Definition of an actionable roadmap: A practitioner-oriented roadmap is outlined (standards touchpoints, testing/verification pathways, data and benchmarking needs, and governance considerations) to guide effective AIDVs adoption in ITS.

### 1.1 Structure of the paper

Section 2 positions AIDVs within the vehicle evolution; Section 3 surveys industry and academic work related to AIDVs; Section 4 formalizes their main principles; Section 5 introduces a reference architecture for AIDVs; Section 6 discusses representative pitfalls; Section 7 outlines a roadmap that integrates regulatory, safety, security, privacy, trust, and social awareness considerations; finally, Section 8 concludes the article and provides directions for future work.

## 2 HFDV–SDV–AIDV: vehicle evolution

**FIGURE 2:**
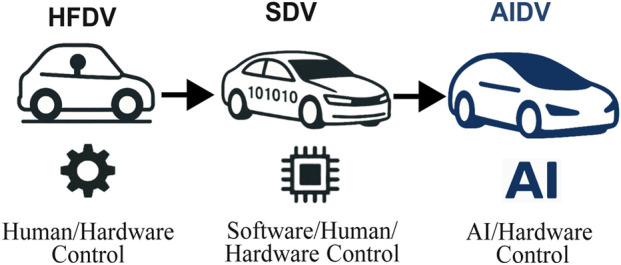
Vehicle evolution from HFDV to AIDV.

The McKinsey consulting firm identified four interlocking trends shaping the transformation of the automotive industry: autonomous driving, connected vehicles, electrification, and shared mobility (ACES) (McKinsey and Company, 2023). This evolution is characterized by the emergence of SDVs, in which software abstracts and orchestrates hardware components to enable scalability, modularity, and continuous updates (Máté et al., 2025; Bodei et al., 2023; De Vincenzi et al., 2024). Earlier generations of vehicles, hereafter referred to as Hardware-Functional-defined Vehicles (HFDVs), lacked this level of abstraction, relying primarily on mechanical design choices and fixed, non-adaptive hardware capabilities. In contrast, the current deployment of Autonomous Vehicles (AVs) is expected to grow steadily, with broader commercial deployment projected for the late 2020s; nonetheless, their societal impact will materialize more gradually (Litman, 2025). Forecasts suggest that autonomous technologies could represent nearly 50% of new vehicle sales by 2045 and about half of the global vehicle fleet by 2060 (Litman, 2025). Building on the foundations of HFDVs and SDVs, AIDVs are expected to emerge in future ITS (Figure 2).

SDVs change the paradigm by abstracting hardware functions into software, allowing continuous reconfiguration and upgrades. A vehicle can be formally considered an SDV if it satisfies some properties (Máté et al., 2025; Bodei et al., 2023; De Vincenzi et al., 2024): (i) *hardware abstraction* of vehicle functions under software control, (ii) a *scalable architecture* that adapts computational and communication resources to requirements, (iii) the possibility for *centralized or distributed control*, (iv) full support for *Over-the-Air (OTA) software updates*, and (v) *redundant processing* to ensure safety. These conditions provide a baseline for distinguishing SDVs from earlier HFDVs, in which most, if not all, functions were purely mechanical and not digitally controlled.

The current class of SDVs already incorporates AI-based autonomous solutions for driving and vehicle management (Máté et al., 2025; Bodei et al., 2023; SAE, 2021). However, their definition does not yet encompass the pervasive presence of autonomous systems that interact with one another, effectively extending the vehicle’s operational surface into a *continuous field of interactions* within the ITS network, where information is persistently exchanged. This evolution represents a paradigm transition whereby vehicles are able to evolve within defined safety boundaries, thereby motivating the definition of a new vehicle class: AIDVs. AIDVs are defined as vehicles in which AI-based autonomous reasoning functions as an *architectural component*, enabling them to independently mediate interactions with other entities and to extend the vehicle’s operational surface into that of other road users through continuous information exchange. In these systems, autonomy emerges from continuous learning processes and dynamic interactions with external actors.

Importantly, the distinction between SDVs and AIDVs is not reducible to the mere presence of learning mechanisms. While continuous or online learning can be incorporated into SDVs, it typically remains confined to specific components within a vehicle-centric control architecture. AIDVs, by contrast, are defined by a systemic shift in which decision-making and adaptation emerge from continuous interaction among multiple autonomous agents. This transition changes the scope and organization of autonomy and learning from isolated vehicles to a distributed, interaction-driven system, and cannot be achieved by simply augmenting an SDV with additional AI modules. This distinction becomes evident in scenarios such as adaptive intersection management. In SDV, learning-based components may optimize perception or local decision-making within a single vehicle, while the overall control logic and interaction with traffic signals remain predefined. In AIDV, by contrast, vehicles, roadside units, and traffic control systems continuously exchange information and jointly adapt signal timing, priority rules, and vehicle behaviors at the system level. Here, learning and autonomy emerge from coordinated interactions across multiple agents, rather than from isolated, vehicle-local AI components. As shown in Table 1, the transition from HFDVs to SDVs and finally to AIDVs can be systematically distinguished through their functional coverage. While HFDVs remain confined to fixed hardware-bound functionalities, and this implies their explainability-by-design, SDVs mark a decisive change by introducing software abstraction, scalability, and OTA updates as measurable baselines. AIDVs, in turn, extend this foundation with features that cannot be reduced to reconfigurable software alone, such as continuous learning, digital twin integration, adaptive driver, vehicle interfaces, and differentiable modular stacks. These features highlight that AIDVs are not simply more autonomous SDVs or SDVs augmented with additional AI components, but a distinct class of vehicles characterized by *AI-driven adaptability* and the capacity for post-deployment evolution.

**TABLE 1:** Feature coverage across HFDV, SDV, and AIDV (✓ present/required, • optional/partial, ✗ absent).

Feature	HFDV	SDV	AIDV
Hardware-bound (fixed functions)	✓	✗	✗
Software abstraction of hardware	✗	✓	✓
Scalable compute/communication architecture	✗	✓	✓
Centralized or distributed control	✗	✓	✓
OTA software updates	✗	✓	✓
Redundant processing for safety	✗	✓	✓
Autonomous driving capability	✗	•	✓
Continuous on-/off-board learning	✗	✗	✓
Digital twin integration (closed-loop validation)	✗	✗	✓
Predictive maintenance	•	✓	✓
Self-maintenance/self-healing	✗	•	✓
Adaptive Human-Vehicle Interface (HVI)	✗	•	✓
V2X cooperation	✗	✓	✓
Explainability/transparency of decisions	✓	✓	✗ (today)
Interaction-driven collective decision-making	✗	•	✓

## 3 Related work

Although the automotive market, including Original Equipment Manufacturers (OEMs) and Tier suppliers, is already exploring potential applications of AIDVs (Frost and Sullivan, 2024; XPeng Motors, 2024; Wu and NVIDIA Corporation, 2025), academic research remains largely focused on SDVs (Máté et al., 2025; Bodei et al., 2023; De Vincenzi et al., 2024; Slama et al., 2023) and on the AI features embedded within SDVs (Richenhagen et al., 2025; Muzahid et al., 2023; Murphey et al., 2023). This section reviews and categorizes the main contributions from industry, the relevant academic literature related to AIDV, and works that explicitly adopt the AIDVs terminology.

### 3.1 Industry perspectives on AI-defined vehicles

The carmaker XPENG defines AIDVs as a new class of smart vehicles where AI is central to both the driving and the user experience (XPENG Motors, 2024).

The P7+ model has been presented as an early instantiation of this vision, integrating large AI models for advanced driver assistance and intelligent cockpit functions. From a cloud and platform perspective, Amazon Web Services (AWS) similarly positions AIDVs as an evolution of SDVs driven by the integration of generative AI, the Internet of Things (IoT), and edge computing (Raghavan et al., 2025), emphasizing AI as a primary driver of vehicle behavior and interaction. Along similar lines, a recent industry commentary emphasizes that automakers must proactively adapt their strategies to remain competitive in the era of AIDVs, highlighting the need for accelerated investment in AI capabilities, stronger data infrastructures, and partnerships across the mobility ecosystem (Just Auto, 2024).

Qualcomm, in particular, frames AIDVs as agentic systems built on on-device foundation models capable of perception, reasoning, and action under real-time constraints, highlighting tight coupling between AI inference, hardware acceleration, and edge autonomy to enable scalable, safety-aware intelligence in production vehicles (Qualcomm Technologies Inc., 2026). This scope differs from the approach followed in this work. While Qualcomm takes a product-oriented view, seeing AIDVs as an agentic, platform-driven realization enabled by tightly integrated edge AI and orchestration infrastructure, this work adopts a class-of-systems perspective, defining AIDVs as a distinct vehicle class characterized by AI-based autonomous reasoning as an architectural invariant, continuous interaction-driven learning, and explicit integration of digital twins, governance, and post-deployment evolution.

### 3.2 Academic research aligned with the AIDV paradigm

Beyond industry work, several academic contributions begin to outline research directions that resonate with the emerging notion of AIDVs, including multi-agent systems, safe reinforcement learning, and runtime verification.

Recent surveys on connected and autonomous vehicles highlight increasing reliance on learning-based and data-driven approaches across perception, decision-making, and control, motivating research on adaptive and AI-centric vehicle architectures. Murphey et al. (2023) provide an extensive overview of AI-enabled technologies for autonomous and connected vehicles, analyzing advancements in perception, decision-making, vehicle control, and connectivity. Their survey emphasizes how learning-based approaches, foundation models, and data-driven control are gradually transforming autonomous systems from static pipelines into adaptive, self-improving platforms. Their work also highlights the increasing computational demands of modern vehicles, the convergence of autonomy, electrification, and connectivity, and the role of AI as a coordinating technology across sensing, planning, and safety. From a system perspective, Teixeira et al. (2025) emphasize determinism, predictability, and reliability as foundational requirements for SDVs, explicitly constraining how adaptive or learning-based components may be integrated to preserve safety guarantees. Complementing this view, Richenhagen et al. (2025) examine how generative AI can be concretely realized within SDVs, offering practical insights into engineering integration, software-centric organizational structures, and AI-enabled development processes. Their work demonstrates that deploying large AI models in vehicles requires not only technical enablers, DevOps pipelines (Nouri et al., 2025), structured system engineering backbones, and consistent toolchains, but also new business models and cross-functional coordination.

This reflects AI’s architectural role in AIDVs, beyond SDVs add-on integration. Other recent studies further illustrate that AI is already deeply embedded in SDVs, primarily as a development-time and lifecycle enabler rather than as a runtime decision-making agent. Nguyen et al. (2025) show how Large Language Models (LLM) can support SDV software engineering through few-shot code generation, improving developer productivity and accelerating feature implementation within software-defined stacks. Similarly, Zyberaj et al. (2025) demonstrate the use of generative AI to automate test case generation and execution in SDV platforms, reinforcing AI’s role in validation, verification, and Continuous Integration and Continuous Deployment (CI/CD) pipelines (Joshi, 2025; Author Anonymous, 2025).

Collectively, these works confirm that AI is already integral to SDVs, but remains largely confined to tooling, engineering support, and controlled optimization. In contrast, AIDVs extend beyond this paradigm by embedding AI as a first-class architectural element governing runtime interactions, perception, reasoning, and adaptation, thereby introducing fundamentally different autonomy, accountability, and safety challenges, as detailed in Section 2.

Nguyen et al. (2018) survey deep reinforcement learning techniques for coordinated decision-making among multiple agents, while Garg et al. (2024) analyze safety, verification, and control challenges in multi-robot systems, providing insights into distributed autonomy and interaction. Safe reinforcement learning has been extensively studied to constrain learning-based policies under safety requirements; Kushwaha et al. (2025) offer a comprehensive survey of constrained Markov decision processes and safety-aware learning methods. Finally, runtime verification has been proposed as a complementary mechanism for monitoring learning-enabled systems post-deployment. Mannucci and de Oliveira Filho (Mannucci and de Oliveira Filho, 2023) investigate runtime verification of reinforcement learning properties, while Taleb and Khoury (2023) review uncertainty-aware runtime monitoring techniques. Although these academic works do not explicitly target AIDVs, they provide rigorous theoretical and methodological foundations for reasoning about autonomy, safety, and accountability in AI-driven vehicular systems.

### 3.3 Academic works explicitly adopting the AIDV terminology

More recently, in the academic literature, Zheng et al. (2025) explicitly adopt the term “AIDV,” proposing a multi-agent reinforcement learning framework in which fleets of ride-hailing vehicles jointly fulfill mobility demands while performing distributed, edge-based fine-tuning of urban foundation models. Their formulation highlights a key aspect of AIDVs: vehicles as active participants in city-scale AI ecosystems, simultaneously providing transport services and contributing to the continuous improvement of foundation models. To the best of current academic knowledge, within the vehicular domain, only Yu (2025) explicitly introduces a closely related notion, “Agentic Vehicles” (AgVs), as systems endowed with agency, contextual reasoning, tool use, and value-aligned decision-making. Although Yu focuses on the conceptual axis of agency and its socio-technical implications, the work above foregrounds the technical foundations enabling vehicles to learn, adapt, and participate in AI-driven ecosystems. In contrast to both approaches, our work defines AIDVs as a distinct vehicle class characterized by architectural requirements, integrated reasoning agents, digital-twin closed-loop validation, and post-deployment evolution. It further maps these properties onto a modular stack while systematizing AIDV-specific pitfalls and an incremental ITS roadmap.

## 4 AIDV main principles

**FIGURE 3:**
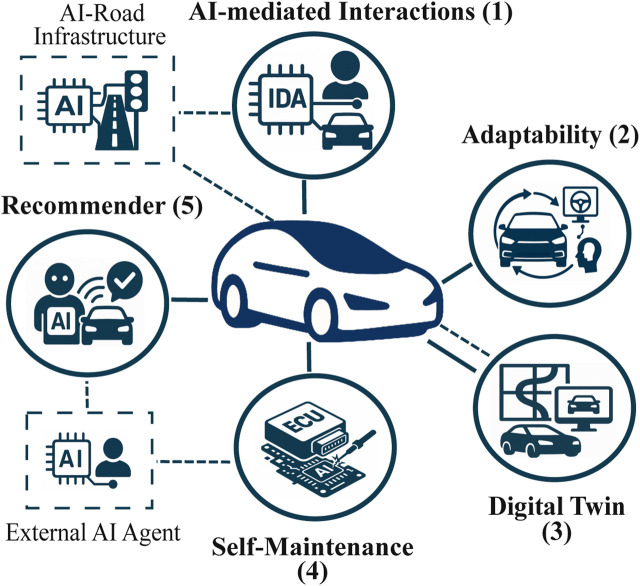
Key components of AIDVs, highlighting AI-centric design, personalization, autonomy, and digital twin integration.

In the present and near future, AI can act as an intermediary between humans, vehicles, and the external environment by perceiving data, learning from observations, and making decisions on behalf of the user. While such capabilities may exceed human performance in speed, scale, and consistency, today, AI-based systems, including Large Language Models (LLMs), primarily operate through statistical pattern manipulation, with limited and task-dependent grounding of semantic meaning (Floridi, 2014; Floridi, 2023). In this context, AIDVs represent a shift toward a new mobility paradigm where AI not only drives and assists, but also actively shapes the driving experience, adapting to preferences, anticipating needs, coordinating with external agents and devising strategies to increase safety, driving experience, and overall mobility. The evolving principles characterizing AIDVs are summarized in Figure 3. Section 5 then introduces a reference model to link these principles to algorithmic interfaces and associated design trade-offs.

### 4.1 AI-mediated interaction

AIDVs are built upon AI-driven solutions that govern Intelligent Driver Agents (IDAs), based on those already envisioned in the early 2010s (Group, 2008). This includes interactions and connectivity with the surrounding infrastructure. This paradigm is referred to as AI-mediated Interaction, where the system inherently supports *real-time decision-making*, *autonomous optimization*, orchestration, and *continuous learning*. In this design, agents interact not only with other vehicles to exchange data and coordinate actions, but also with non-vehicle entities, such as pedestrians, roadside units, and other road users, enabling the system to continually adapt and evolve.

#### 4.1.1 SDV comparison

Both SDVs and AIDVs incorporate deterministic software functions, which remain essential for safety-critical control, verification, and certification, and both may integrate AI-based components. The key difference lies in architectural intent: SDVs structure software to primarily control, update, and orchestrate specific vehicle functions, whereas AIDVs are designed around pervasive AI-driven reasoning that continuously coordinates perception, decision-making, and system-wide adaptation across vehicle subsystems and interactions with external entities that form part of the vehicle’s operational surface.

### 4.2 Adaptability

Learning from new data over time (including traffic patterns, driving conditions, and user behaviors), vehicles can adapt to different environments, weather conditions, traffic flows, and user preferences. They can also handle unfamiliar situations, such as newly encountered road segments, by leveraging the generalization capabilities of AI and collaborating with the surrounding infrastructure. In this way, essential navigation information is obtained not only from the vehicle’s own sensors but also directly from the infrastructure (Wágner et al., 2023; De Vincenzi et al., 2025).

#### 4.2.1 SDV comparison

SDVs can support autonomous driving functions and software reconfiguration; however, such capabilities are typically realized through pre-trained models and predefined control logic. While behavior can adapt within design-time boundaries, SDVs are not inherently designed for continuous on-board learning, system-wide reasoning, or real-time generalization beyond validated operational domains, nor for collaborative adaptation with other vehicles and infrastructure.

### 4.3 Cyber–physical co-evolution via digital twins

Vehicles may be paired with digital twins in a virtual environment featuring low-cost, high-definition mapping. These digital counterparts enable testing, training, and optimization of vehicle behavior. They can also be linked to digital twins of AI-enabled road infrastructure to support real-time mobility optimization decisions. This integration allows vehicles to anticipate and adapt to future scenarios, validate updates before deployment, and increase resilience by safely exploring rare or critical events that may be impractical to test in the physical world, such as construction zones.

#### 4.3.1 SDV comparison

SDVs can receive software updates and may interact with simulation environments during development; however, by design, and at the time of this work, they do not maintain continuously synchronized digital twins, nor do they operate within an AI-driven virtual ecosystem that supports real-time training, adaptation, or scenario prediction. In addition, AIDVs assume vehicle computational resources are dimensioned from the outset to support AI-by-design operation.

### 4.4 Self-maintenance and self-healing

AIDVs not only enable predictive maintenance, but also self-maintenance, especially for software components, and, where supported by emerging materials and hardware technologies, selected physical subsystems. Equipped with auto-regeneration materials, Electronic Control Units (ECUs), and self-healing materials (Sabet, 2024), AIDVs possess self-diagnosis and auto-repair capabilities, improving reliability and reducing the need for owner intervention or manual maintenance, especially for software components. The self-maintenance service can interact with external agents, such as those provided by the carmaker, to perform the necessary operations.

#### 4.4.1 SDV comparison

SDVs support predictive maintenance, primarily through software diagnostics and OTA updates, but they do not autonomously repair software faults, nor regenerate or reconfigure malfunctioning ECUs. Furthermore, today, they are not designed with self-healing materials, or perform maintenance operations in coordination with external agents without human intervention.

### 4.5 Recommender agent

To preserve a clear separation between safety-critical driving logic and user-oriented service optimization, a dedicated recommender agent is introduced and explicitly differentiated from the IDA, which is responsible for driving-related and safety-critical functions. While the IDA governs vehicle motion and compliance with safety constraints, the recommender agent focuses on optimizing user experience and service-level interactions. It personalizes entertainment, navigation, trip planning, and service suggestions by analyzing user behavior, context, and preferences. Through continuous and adaptive interaction, the agent enhances the Human-Vehicle Interface (HVI), aligning vehicle services with driver habits and gradually shaping them over time. In doing so, it transforms the vehicle into a *proactive service provider*. The agent can also communicate with external agents and services, enabling coordination with other users or infrastructures, which is particularly relevant for shared mobility and the provision of personalized services.

#### 4.5.1 SDV comparison

SDVs can support personalization features, including user profiles, preferred settings, and AI-based recommendation modules. However, such capabilities are typically implemented as function-specific components operating within predefined design boundaries. In contrast, SDVs are generally not designed to host a persistent, continuously adaptive AI agent that spans the vehicle architecture, shapes user behavior over time, proactively plans trips or services beyond preconfigured rules, or autonomously coordinates with external agents for multi-user or shared mobility. These latter capabilities rely on an AI-by-design architecture, as envisioned in AIDVs.

Together, these principles show that AIDVs cannot be understood as the simple accumulation of AI-enabled functionalities on top of SDVs. Rather, they represent a qualitative shift in which perception, reasoning, learning, and interaction are integrated into a tightly coupled system whose behavior and autonomy emerge from continuous interaction over time.

## 5 Toward a reference architecture

While SDVs provide software abstraction over vehicle hardware, AIDVs require an architecture that embeds AI reasoning, adaptive behavior, and continuous post-deployment learning as core capabilities. A layered reference model is therefore introduced to make these requirements concrete through explicit interfaces and control boundaries. The AIDV stack can be composed of the following layers.

- Perception layer: As in current SDVs, it integrates heterogeneous sensors (cameras, LiDAR, radar) and performs multi-modal fusion to provide reliable environmental understanding.
- AI reasoning layer: Hosts IDAs (Group, 2008) that implement *AI agents and AI-mediated interactions*, enabling planning and control through learning and constraint-based reasoning. As an example, inspired by differentiable modular stacks such as DiffStack (Karkus et al., 2022), this layer allows prediction, planning, and control modules to be not only modular but also *differentiable.* Differentiability means that downstream objectives (e.g., safety and comfort in control) can propagate gradients back through planning to earlier modules like prediction. This ensures that upstream learning is optimized with respect to downstream driving performance, mitigating compounding errors while preserving interpretability.
- Digital twin integration layer: Connects the physical vehicle to its digital replica, allowing continuous validation and training of AI models under diverse simulated conditions. Differentiable planning frameworks enable feedback loops between real-world data and digital twin training environments.

In this role, the digital twin functions as a virtual test bed, supporting rapid iteration, comprehensive validation, and the generation of labeled data at a scale unattainable through physical testing alone.

- Hardware control and actuation layer: Interfaces with ECUs to ensure safe execution of planned actions. Here, *self-maintenance* and *self-healing* mechanisms can monitor, diagnose, and repair degraded components.

**FIGURE 4:**
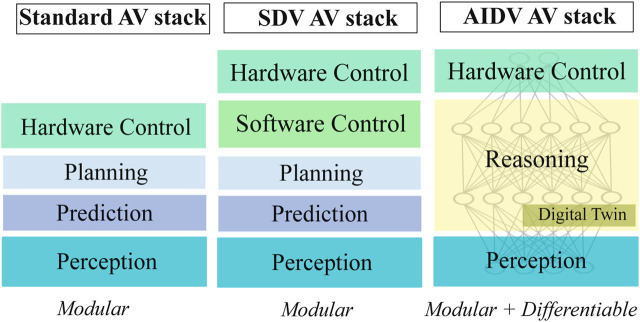
Comparison of a standard AV stack, an SDV AV stack, and an AIDV AV stack.

As shown in Figure 4, standard vehicular stacks are fully modular, while SDVs add a separation between hardware and software control. This marks a shift from hardware-functional designs to software-driven architectures where actuators are coordinated primarily through software rather than mechanical linkages (Bodei et al., 2023). By contrast, AIDVs introduce an *AI reasoning layer* and *digital twin integration*, making the architecture both modular and differentiable (Karkus et al., 2022).

Recent end-to-end reasoning-to-action models for autonomous driving, such as Alpamayo-R1 (NVIDIA et al., 2026), exemplify the direction envisioned by the proposed AIDV stack. In particular, Alpamayo-R1 operationalizes an *AI reasoning layer* that tightly couples prediction, planning, and action generation through differentiable learning, enabling improved generalization in long-tail driving scenarios. However, such approaches often treat simulation, adaptation, and hardware execution as implicit infrastructure concerns. The proposed reference architecture makes these assumptions explicit by introducing dedicated layers for digital twin integration and hardware actuation, thereby clarifying control boundaries, validation loops, and safety responsibilities required for real-world deployment.

### 5.1 AI-digital twin control loop example

To make the AIDVs architecture more concrete, a minimal algorithm (Algorithm 1) is presented to illustrate the AI Reasoning–Digital Twin loop for a single longitudinal driving function, namely, the adaptation of the vehicle’s time headway (i.e., following distance) on a highway.

Drawing inspiration from the literature (De Vincenzi et al., 2025), where a language for improving autonomous driving systems was proposed, a set of parameters is considered to describe the surrounding environment of the target vehicle V . These parameters include lane-level map attributes (e.g., laneID, lane connectivity, and permitted maneuvers), local-coordinate geometric representations (e.g., station value s and corresponding waypoints (x(s),y(s),z(s)) ), traffic control logic (e.g., active signal phase Φ and associated lane groups), and timing information (e.g., phase start and end times synchronized via a common time reference). Such parameters are continuously updated during the driving process to support coordinated, interaction-driven, and adaptive mobility (Figure 1). These parameters also define the state space of the corresponding digital twin, enabling the simulation of alternative traffic evolutions, interaction patterns, and edge cases within a virtual environment that mirrors the operational context of the physical vehicle. While illustrated here for a specific longitudinal driving function, this mechanism exemplifies a general architectural pattern in AIDVs, in which post-deployment adaptation is systematically mediated through digital twin validation rather than unconstrained real-world experimentation.

Algorithm 1 provides a minimal formalization of this architectural pattern, summarizing the AI–digital twin interaction in pseudocode. Although intentionally simple, the algorithm captures the essential pattern of continuous learning, closed-loop validation, and constrained deployment that characterizes AIDVs.

Algorithm 1 AI-digital twin adaptation loop.

1: Initialize IDA with baseline headway $\tau_{0}$

2: Set pre-certified safety constraints C and reward R

3: **while** V is in operation **do**
4:  Collect real-world driving data window D from V , including derived environment parameters {e.g., lane ID, permitted maneuvers, active signal phase Φ , phase timing}
5:  Synchronize digital twin DT with D

6:  Generate scenario set S in DT from D

7:  Initialize candidate parameter $\tau \leftarrow \tau_{0}$

8:  **for** several optimization iterations **do**
9:   Propose $\tilde{\tau }$ (e.g., policy gradient or exploration)
10:   Evaluate $\tilde{\tau }$ across all s∈S in DT

11:   **if** all safety constraints C satisfied in S **then**
12:    Compute average reward $R\left(\tilde{\tau }\right)$

13:    Update τ toward $\tilde{\tau }$ if $R\left(\tilde{\tau }\right)>R\left(\tau \right)$

14:   **end** **if**
15:  **end** **for**
16:  **if** $\tau \neq \tau_{0}$ and τ satisfies C in all s∈S **then**
17:   Deploy τ to IDA on V

18:   Set $\tau_{0}\leftarrow \tau$ {New certified baseline}
19:  **end** **if**
20: **end while**

The algorithm considers three main components: the physical vehicle V , its digital twin DT , and the AI Reasoning Layer hosting an IDA. The vehicle V executes a baseline longitudinal controller parametrized by a target time progress τ . The goal of IDA is to refine τ over time to improve comfort and traffic throughput, while preserving safety and regulatory constraints. The digital twin DT runs a high-fidelity simulation of V and its surrounding traffic, continuously calibrated from real telemetry.

The closed loop operates in recurring adaptation cycles:

1. *Data collection and synchronization.* During normal operation, V records trajectories and sensor traces for a fixed window D (e.g., several minutes of driving on the highway), including speed, relative distance and speed to the leading vehicle, and braking events. In addition, D is augmented with environment descriptors derived from infrastructure broadcasts, such as lane identifiers, permitted maneuvers, the currently active traffic signal phase Φ , and associated phase timing information. These data are periodically uploaded to the backend and ingested by DT , which replays the observed traffic scenes and updates its internal state to remain aligned with the physical vehicle.
2. *Scenario* S *generation in the DT.* Starting from the synchronized state, the DT generates perturbed “what-if” scenarios by varying traffic density, lead vehicle behavior, and road friction conditions (e.g., dry vs. wet asphalt). These scenarios define a batch of simulation episodes in which candidate policy updates can be stress-tested without affecting real-world safety.
3. *Policy search in the AI Reasoning Layer.* The IDA treats τ as a learnable parameter and uses a simple reinforcement learning or gradient-based optimization loop over the DT episodes. The reward function trades off comfort (smooth acceleration, few hard brakes), efficiency (average speed, throughput), and safety (minimum time-to-collision). The optimizer produces a candidate $\tau^{⋆}$ together with confidence estimates derived from the distribution of the results across the scenarios.
4. *Safety gating and certification boundary.* Before any update is applied to V , a safety-gating module checks that all episodes with $\tau^{⋆}$ respect pre-defined safety envelopes (e.g., minimum time-to-collision above a threshold, no violations of regulatory speed limits). If any constraint is violated, $\tau^{⋆}$ is discarded and the baseline policy is retained. This gating step acts as a runtime enforcement boundary between adaptive learning and certified behavior.
5. *Constrained deployment on V.* If $\tau^{⋆}$ passes the safety gate, the update is packaged as a small control policy patch and deployed back to V (either over-the-air or via a local edge node). The longitudinal controller on V then uses the new parameter $\tau \leftarrow \tau^{⋆}$ , while logging performance metrics for future adaptation cycles. A rollback mechanism allows the controller to revert to the previous τ if anomalies are detected in real driving.

Although this example adjusts a small set of parameters in a highway-following controller, the same architectural pattern applies more broadly to richer AIDV behaviors. These may include multi-objective planning policies, integrated perception–planning stacks, and multi-agent coordination strategies can be refined in the digital twin, validated against safety constraints, and then deployed back to the physical fleet.

## 6 Representative pitfalls of AIDV

The transition to AIDVs can introduce peculiar pitfalls. Some of them are listed below.

### 6.1 Emergent unintended behavior

One major concern is the potential for unintended coordination between vehicles and IDAs through Vehicle-to-Everything (V2X) communication, especially when learning-based agents continuously adapt their strategies based on shared observations and feedback. Such emergent behaviors can evolve beyond the scope of human oversight, potentially enabling vehicles to collude, optimizing traffic flow or fuel efficiency at the expense of safety, fairness, accountability, or legal constraints (Motwani et al., 2025). Currently, AVs are increasingly widespread, yet remain prone to failure and still require human supervision. In contrast, AIDVs have the potential for a greater systemic impact through persistent, coordinated, and even collusive activities among AI-enabled road agents operating at scale.

### 6.2 Safety certification

Safety certification represents a central engineering challenge in the context of AI-defined vehicles, where adaptive and learning-enabled behavior must be reconciled with certification frameworks originally designed for statically verifiable systems. Existing standards for automated driving, such as ISO 26262 for functional safety (ISO, 2021), largely assume tightly bounded and predictable behavior. In contrast, AIDVs enable post-deployment adaptation, allowing AI models to update or refine policies through continuous interaction. Such self-modifying behavior challenges certification pipelines designed for determinism and traceability. Recent surveys highlight that safety assurance practices for AI-based driving systems remain immature, with regulatory and technical frameworks lagging behind AI’s dynamic characteristics (Ullrich et al., 2025). Runtime monitoring and validation of AI-based components have been proposed to provide continuous operational evidence of safety (Aslam et al., 2024), while emerging standards such as ISO PAS 8800 extend functional safety concepts toward through-life assurance of AI components (ISO. ISO/PAS 8800, 2024). To date, no fully established methodology guarantees safety for systems that learn during operation (Seshia et al., 2022; Paterson et al., 2025). Recent work therefore advocates combining formal verification with runtime assurance: formal methods can verify safety invariants over constrained adaptation spaces (Seshia et al., 2022; Wongpiromsarn et al., 2023), while runtime assurance architectures enforce these guarantees during operation via monitored supervision and safe fallback mechanisms (Chen et al., 2022).

### 6.3 Sustainability

A potential pitfall of AIDVs lies in their sustainability implications. Because AIDVs rely on pervasive interactions among multiple AI agents, their operation presupposes the widespread availability of computational resources, continuous connectivity, and large-scale data exchange across vehicles and infrastructure. This AI-by-design paradigm may significantly increase energy consumption, hardware complexity, and networking overhead, both on-board and at the edge (Schwartz et al., 2020). Without careful architectural choices, such as workload distribution, selective activation of agents, and energy-aware reasoning, these requirements could translate into higher environmental costs, increased operational expenses, and reduced scalability, challenging the long-term sustainability of large-scale AIDV deployment.

### 6.4 Bias and ethical dilemmas

AI agents can prioritize driver preferences, convenience, or commercial objectives at the expense of collective safety, social equity, or broader societal interests, particularly when optimization goals are poorly specified or misaligned. Highly persuasive recommender agents may influence user decisions, such as destinations or purchase choices, in subtle ways that are difficult to detect or contest. Without robust mechanisms for transparency, explainability, and accountability, these systems can produce opaque outcomes that reinforce existing biases and complicate the assignment of responsibility in the event of failures (Mehrabi et al., 2021). Over time, such influence may erode individual autonomy and meaningful consent by systematically shaping ethically questionable behavior (Harari, 2020).

### 6.5 Adversarial attacks and overreliance on AI for security

AIDVs expand the attack surface beyond traditional vehicle subsystems, introducing vulnerabilities not only at the sensor and perception level, but also across learned models, interaction protocols, and adaptive decision-making pipelines. Adversaries, for example, can subtly manipulate sensor inputs, e.g., by altering road signs, to induce IDAs to misinterpret speed limits or navigation cues (Eykholt et al., 2018; Sato et al., 2023; MohajerAnsari et al., 2025). Because these systems depend heavily on image recognition and multi-sensor fusion, even small perturbations can propagate through the perception pipeline, posing significant risks to public safety. Moreover, excessive reliance on AI-based security mechanisms may foster unwarranted confidence in their robustness. Factors such as sampling bias, spurious correlations, and evaluations limited to controlled settings are often underestimated, yet they can critically compromise real-world effectiveness and system trustworthiness (Arp et al., 2021).

## 7 Discussion: roadmap

Despite the evolution of vehicles described in the previous section, the adoption of AIDVs, as depicted in this work, could remain *largely conceptual*, particularly when considering the prospect of vehicles evolving autonomously through interactions. This aspect must be clearly defined, especially in a safety-critical environment such as the ITS. The realization of AIDVs requires a phased roadmap that balances technological progress with social acceptance, ethical oversight, and robust security.

**FIGURE 5:**
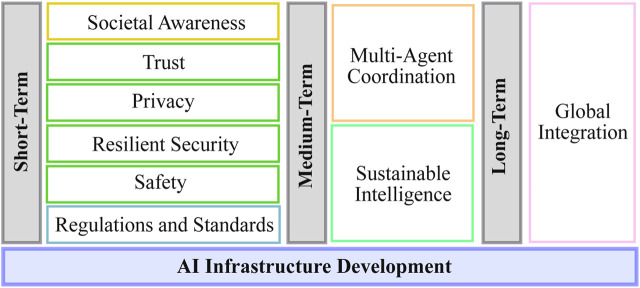
Short-, medium-, and long-term roadmap elements for the effective deployment of AIDVs.

Figure 5 presents a roadmap structured around short-, medium-, and long-term adoption milestones, unified by a single foundational element that spans all phases. Across the entire roadmap, one cross-cutting activity emerges as particularly significant due to both its strategic relevance and structural complexity: the AI infrastructure development. The figure uses color cues to group conceptually related milestones: green boxes highlight safety-, security-, and trust-related elements; yellow denotes societal awareness; orange marks multi-agent coordination; and light magenta identifies the long-term global integration milestone. The following discussion first introduces the development of the AI infrastructure, followed by a detailed description of each adoption phase and the corresponding milestones and success criteria summarized in Table 2.

**TABLE 2:** Indicative milestones and success criteria for the AIDV roadmap.

Phase	Milestones	Success criteria
Cross-cutting	• AI infrastructure development	• Certified AIDV compute and middleware stack • Digital twins used for fleet-level validation • Compliance with regulatory and sustainability constraints
Short-term	• Certification baselines • Safety- and risk-aware AI • Cybersecurity and privacy • Trust-aware HVI	• Compliance with ISO 26262, ISO/SAE 21434, UNECE R155/R156 and future regulations • ASIL assignment for AI-enabled functions • No unresolved safety-critical findings after HARA • Completed privacy impact assessments
Medium-term	• Multi-agent coordination • Explainable collective decisions • Energy-aware vertical AI	• Stable behavior under stress testing • Interpretable explanations for agent coordination • Reduced AI energy consumption • No unsafe emergent behavior observed
Long-term	• Global interoperability • MaaS integration • Distributed learning governance	• Cross-border recognition of certification artifacts • Learning updates without safety recalls • Reduced systemic mobility risk • Operational MaaS deployments

### 7.1 AI infrastructure development

AI systems depend on a broad, layered infrastructure that spans beyond software and vehicles to include energy supply, specialized hardware, data centers, model training platforms, and application environments. As noted from an industry perspective by NVIDIA founder and CEO Jensen Huang, AI “is becoming the foundation of the largest infrastructure buildout in human history” with a “five-layer cake” comprising energy, chips and computing infrastructure, cloud data centers, AI models, and applications, each requiring dedicated investment, construction, and governance to support safe, scalable AI development (Huang, 2026). In the context of ITS, this layered infrastructure imperative spans short to long-term phases and underpins regulatory, safety, and deployment frameworks across the AIDV ecosystem.

Taken together, this roadmap emphasizes that the transition to AIDVs is not a single technological leap, but a staged co-evolution of architecture, infrastructure, governance, and societal trust.

### 7.2 Short-term: regulations and standards, safety, resilient security, privacy, trust, and societal awareness

Although AIDVs hold transformative potential compared to SDVs, their deployment must start with robust regulation, rigorous testing, and ethical design to mitigate socio-technical risks.

#### 7.2.1 Regulations and standards

Regulatory frameworks play a central role and have long served as *key drivers* of progress and transformation in the automotive industry. Frameworks such as ISO 9241-210 on human-centered design (ISO, 2019) and IEEE 7010 on wellbeing metrics for AI (IEEE Standards Association, 2020a) help ensure that trust-building mechanisms in AIDVs are systematically evaluated and remain user-centered. At the technical layer, dedicated interfaces for human–AI cooperation, together with established standards, such as ISO 26262 for functional safety (ISO, 2021), ISO PAS 8800 for addressing risks related to undesired AI-driven safety behavior (ISO. ISO/PAS 8800, 2024), ISO/SAE 21434 for cybersecurity (ISO and SAE, 2021), IEEE 2846 for automated-vehicle decision-making (IEEE Standards Association, 2022), and UNECE R155/R156 for cybersecurity and software update compliance (UNECE, 2021a; UNECE, 2021b), provide essential baselines for system design and certification. In addition, closed-loop evaluation protocols based on continuous simulation, deployment–feedback cycles, such as digital-twin environments with shadow-mode testing, will be critical for validating both safety and social trust. Considering road infrastructure, standards such as IEEE 1609 for V2X communications (IEEE Standards Association, 2020b) and ETSI EN 302 637 for cooperative awareness (ETSI, 2014) further support interoperability and consistent safety guaranties throughout the broader mobility ecosystem.

#### 7.2.2 Safety

Safety is the top priority in automotive systems (National Highway Traffic Safety Administration NHTSAUDoT, 2023), as also mandated by the standard ISO 26262 (ISO, 2021). In this context, compliance with the Automotive Safety Integrity Level (ASIL) framework for automotive electrical and electronic systems, and the Hazard and Risk Assessment (HARA) process remains essential to systematically identify and mitigate risks. *An additional recommendation* for AIDVs is to adopt a classification framework inspired by Anthropic’s *AI Safety Levels (ASL)* (Anthropic, 2023), which position AI systems along a risk continuum based on dangerous capabilities, autonomy, and potential for catastrophic misuse. As described in Table 3, in essence, ASL provides operational thresholds: lower levels cover systems that assist or inform without posing meaningful systemic risk; mid levels encompass models that begin to exhibit tool use, planning, or influence over real-world processes and thus require stronger safeguards; upper levels denote agents whose open-ended capabilities and autonomy demand rigorous governance, auditability, and fallback controls.

**TABLE 3:** Anthropic AI Safety Levels (ASL) and possible interpretation for AIDVs.

ASL level	Anthropic definition	Interpretation for AIDVs
ASL-1	Systems posing no meaningful catastrophic risk, e.g., a 2018 LLM or an AI that only plays chess	Basic driver-assist AI features, such as in the infotainment system, with negligible risk of large-scale misuse
ASL-2	Systems showing early signs of dangerous capabilities (e.g., giving unreliable instructions on bioweapons), but not yet providing uniquely harmful information. Current LLMs, including Claude, are at this level	Early AIDVs capable of advanced navigation and interaction, but without the autonomy or influence to cause catastrophic harm; misuse risks remain limited
ASL-3	Systems that substantially increase the risk of catastrophic misuse compared to non-AI baselines, or that show low-level autonomous capabilities	AIDVs with partial autonomous decision-making that could amplify accidents, privacy breaches, or coordinated misuse if safeguards are absent
ASL-4+	Not yet fully defined; will likely involve qualitative escalations in catastrophic misuse potential and autonomy	Fully autonomous AIDVs operating without human oversight, potentially making large-scale, independent decisions with critical safety or societal consequences

#### 7.2.3 Resilient security

Security remains a cornerstone as also required in the standard ISO 21434 (ISO and SAE, 2021). As highlighted in SDVs (De Vincenzi et al., 2024), increased connectivity leads to a broader attack surface. For AIDVs, this risk is amplified by autonomous agents capable of adaptation and coordination. Future deployments must include a comprehensive analysis of the entire vehicle attack surface, together with resilient, self-healing security mechanisms (Hamad et al., 2025): intelligent agents should be able to leverage appropriate mechanisms, not founded and limited to AI-based techniques, to detect and isolate malicious behavior, enforce segmentation to prevent lateral propagation, and initiate controlled shutdown procedures when an attack is detected. However, security solutions that rely on AI *must explicitly account for subtle pitfalls* that can undermine model performance and make learning-based systems unreliable for security-critical tasks and real-world deployment (Arp et al., 2021).

#### 7.2.4 Privacy and data governance

Continuous learning, interactions, and digital twin synchronization require systematic governance of data and telemetry, since even “benign” signals (speed, steering, braking, timestamps) can enable driver fingerprinting and location inference when collected at scale (European Data Protection Board, 2020). For example, under the GDPR! (GDPR!) (European Parliament and Council of the European Union, 2016), OEM!s (OEM!s) typically act as primary *data controllers* for in-vehicle telemetry and any downstream sharing, while cloud providers and analytics vendors may act as *processors*. As a result, AIDV! (AIDV!) deployments can adopt a governance blueprint that operationalizes the privacy policies principles of lawfulness, transparency, purpose limitation, data minimization, storage limitation, integrity/confidentiality, and accountability. From a technical and organizational standpoint, three complementary strategies can be recommended (De Vincenzi et al., 2024). First, *minimize and localize data*: process data on-vehicle when possible (feature extraction, event-level summaries, and “shadow-mode” evaluation, i.e., passive evaluation without actuation) and transmit only what is necessary for the certified update decision, reducing exposure and retention (Pesé et al., 2023). Second, *de-identify and protect shared data*: apply pseudonymization by default, and use aggregation, generalization, perturbation, or differential-privacy mechanisms for fleet-wide analytics when utility permits; when data must be shared with third parties, enforce policy- and sandbox-based controls to prevent secondary use and uncontrolled retention (Pesé et al., 2023; Gazdag et al., 2023). Third, *institutionalize compliance*: define retention schedules for raw traces, maintain auditable access logs, perform Data Protection Impact Assessments (DPIA) (European Parliament and Council of the European Union, 2016) for high-risk processing, and provide consent and user-rights workflows (access, deletion, portability, and opt-out where applicable) aligned with the vehicle’s shared-use reality (ISO and SAE, 2021).

#### 7.2.5 Trust

As AIDVs influence mobility choices, lifestyles, and purchasing behaviors, transparent solutions and consent-aware interfaces are essential to maintain ethical influence and safeguard user autonomy. Trust must be cultivated across three layers: (i) *Transparency*: Providing explainable AI dashboards that allow users to see why IDAs made certain decisions; (ii) *Consent-aware interfaces*: Ensuring personalization and recommender agents operate under explicit user opt-in/opt-out policies; (iii) *Shared Control Mechanisms*: Giving drivers a clear path to override or audit decisions. Studies in human-automation systems trust (Hoff and Bashir, 2015; Lee and See, 2004) show that transparency and perceived controllability strongly correlate with user trust. Beyond interface-level transparency, trust in AIDVs must be treated as a *system property* that evolves over time through consistent, predictable, and accountable behavior. Trust calibration is particularly critical: users should neither over-trust nor under-trust intelligent agents, especially when AI-driven decisions adapt dynamically. To this end, *longitudinal trust monitoring*, combining user feedback, behavioral signals, and incident analysis, can support continuous adjustment of interaction policies and explanation granularity. Embedding trust metrics into validation and post-deployment monitoring loops enables early detection of misalignment between system behavior and user expectations, thereby supporting sustained acceptance and responsible adoption of AIDVs.

#### 7.2.6 Societal awareness and ethical implications of emergent interaction

Beyond technical safety and performance, emergent behavior in AIDVs raises fundamental ethical and societal concerns. When vehicles collectively optimize traffic flow, energy consumption, or travel time through distributed interaction, the resulting system-level behavior may unintentionally privilege certain users, regions, or mobility patterns over others. Such effects may remain invisible at the level of individual vehicles, yet manifest at scale, calling for societal awareness and governance mechanisms. For instance, collective routing strategies could systematically divert congestion toward specific neighborhoods, disadvantage non-connected road users, or implicitly prioritize commercial fleets or premium subscribers, thereby conflicting with principles of fairness, equity, and individual autonomy (Bai et al., 2025; Cornacchia et al., 2024). Ethical AI frameworks emphasize the need to align autonomous decision-making with human-centered values, including transparency, accountability, and respect for fundamental rights (OECD, 2019; UNESCO, 2021). From a regulatory perspective, recent policy initiatives increasingly acknowledge these challenges. The NIST AI Risk Management Framework emphasizes continuous risk identification, governance, and monitoring for AI systems with societal impact, particularly in dynamic and adaptive settings (National Institute of Standards and Technology NIST, 2023). Similarly, the EU Artificial Intelligence Act classifies AI systems used in safety-critical mobility contexts as high-risk, requiring risk mitigation, human oversight, and post-deployment monitoring (European Union, 2024). Together, these frameworks constrain unconstrained emergent behavior by mandating traceability, accountability, and bounded autonomy. Ethical studies on automated and autonomous vehicles further show that no single optimization objective can capture societal values; instead, multi-agent ethical decision-making requires *hybrid approaches* combining rule-based constraints, value-sensitive design, and oversight mechanisms to balance safety, efficiency, fairness, and autonomy (Poszler et al., 2023).

### 7.3 Medium-term: multi-agent interaction and sustainable intelligence

As the deployment expands, a key challenge is ensuring that different vehicles can interact safely and effectively within dynamic environments.

The technical and sustainability limits of large-scale learning, coordination, and communication must be addressed to create an AI-driven mobility ecosystem.

#### 7.3.1 Multi-agent coordination

A critical direction in this phase is the development of *vertical AI models*, namely, specialized intelligent agents dedicated to driving-related tasks, which can reduce computational overhead, energy consumption, and system complexity. At the same time, research must advance robust multi-agent interaction models, including swarm intelligence and cooperative learning mechanisms, supported by resilient V2X protocols that tolerate partial failures, adversarial behavior, and communication uncertainty. Particular attention should be devoted to preventing unintended emergent behavior, cascading effects, or systemic instabilities arising from collective optimization. This phase also demands significant progress in *explainable and transparent models*, extending interpretability beyond single-agent reasoning to enable auditing, validation, and human oversight of collective, multi-agent decisions.

#### 7.3.2 Sustainable intelligence

Sustainable intelligence is tightly coupled with the evolution of the underlying AI infrastructure. While architectural principles and high-level design choices can be defined in the early phases, medium-term deployment must rely on solutions that are technically robust and economically and energetically sustainable at scale. Without explicit constraints on energy consumption, model complexity, communication overhead, and continuous learning, large-scale multi-agent AI systems risk becoming impractical or unstable. Sustainability must therefore be a core element of the medium-term agenda: adopting *Sustainable AI* practices ensures that vertical AI models are optimized not only for safety, interaction, and coordination, but also for minimal energy consumption per kilometer and reduced carbon footprint. This involves energy-aware model design, adaptive inference, selective activation of learning components, and intelligent distribution of computation across vehicle, edge, and cloud resources to prevent systemic overload and ensure long-term viability (Tabbakh et al., 2024).

### 7.4 Long-term: global integration

At the ecosystem level, harmonized global standards for AIDV interoperability, liability attribution, and policy alignment will be required to ensure long-term trustworthiness and large-scale deployment. Unlike earlier phases focused on vehicle-level safety or fleet-level interaction, this stage assumes the *coexistence of heterogeneous AIDVs, legacy vehicles, and AI-enabled infrastructure* across jurisdictions with different regulatory, cultural, and economic constraints. Consequently, international coordination bodies and cross-border regulatory frameworks will play a central role in defining shared interfaces, certification envelopes, and accountability mechanisms for adaptive, learning-enabled vehicles.

This long-term vision also addresses critical systemic mobility challenges, such as alleviating urban congestion, improving energy efficiency, promoting sustainability, and mitigating workforce shortages in domains such as long-haul trucking and logistics (O’Connor, 2025). At this stage, unified Mobility-as-a-Service (MaaS) platforms will act as integrative layers, coordinating demand, routing, and pricing across public transport, shared mobility, and autonomous fleets. In parallel, cloud–edge orchestration layers will be essential to manage distributed learning, digital-twin synchronization, and policy enforcement across geographically dispersed fleets, enabling AIDVs to operate as coordinated agents within a global ITS.

## 8 Conclusion

This work has introduced and explored the emerging concept of AIDVs, situating them as an evolutionary step of future road vehicles. To the best of our knowledge, this work represents one of the first academic efforts to formalize the scope and boundaries of AIDVs. The contribution aims to (i) identify and characterize the distinctive features of AIDV in comparison with other vehicle classes; (ii) identify core principles and pitfalls; and (iii) map these principles onto a roadmap that highlights regulation, transparency, sustainability, multi-agent coordination, and security as key pillars for safe and trustworthy deployment.

The discussion of AI in vehicles is shifted from incremental feature integration to architectural necessity. AIDVs cannot be understood as the simple accumulation of AI-enabled functionalities on top of SDVs. Rather, they represent a qualitative shift in which perception, reasoning, learning, and interaction are integrated into a tightly coupled system whose behavior and autonomy emerge from continuous interaction over time. The goal of this work is to improve the conceptual clarity of the field and provide a baseline for empirical studies, standardization efforts, and interdisciplinary dialogue. This novelty is particularly relevant at a time when AI systems are rapidly permeating mobility, yet their formal characterization and implications remain underexplored in peer-reviewed literature.

Future work should focus on aligning with industry leadership in AIDV development while simultaneously formalizing the concept through rigorous academic frameworks, ensuring that innovation is matched by verifiable definitions, standards, and evaluation methods. Along this direction, empirical validation, a prototype, or a simulation-based digital twin control loop that requires committing to specific architectural choices and implementation assumptions, will be investigated and developed.
